# The effect of home-based yoga exercises on quality of life among postpartum women

**DOI:** 10.1186/s12978-026-02357-2

**Published:** 2026-06-13

**Authors:** Sally Abd-Elrahman Mohammed, Madiha Hassan, Zeinab Gazar, Mohamed Fayek, Doaa Mostafa Sheashaa

**Affiliations:** 1 Obstetric and Gynecological Nursing, Faculty of Nursing, Delta University for Science & Technology, Gamasa, Egypt; 2 Department of Nursing, Al-Ghad College for Applied Medical Science, KSA, Jeddah, Saudi Arabia; 3 Mansoura University, Mansoura, Egypt; 4 Community Nursing, Faculty of Nursing, Fayom University, Fayom, Egypt; 5 Sport Recreation, Faculty of Physical Education, Mansoura University, Mansoura, Egypt

**Keywords:** Emotional health, Home-based intervention, Maternal health, Physical recovery, Postnatal care, Postpartum period, Quality of life, Women's well-being, Yoga exercises

## Abstract

**Background:**

The postpartum period is often accompanied by physical discomfort, emotional stress, and reduced quality of life. Yoga has gained attention as a gentle, low-cost intervention that can support physical recovery and emotional well-being after childbirth. By combining movement, breathing, and relaxation, postpartum yoga may help improve overall health and quality of life for new mothers.

**Aim of this study:**

To evaluate the effect of a home-based yoga exercise program on the quality of life among postpartum women.

**Design:**

A quasi-experimental design with two groups (study and control).

**Sample:**

A purposive sampling of 300 participants was equally divided.

**Setting:**

This study was conducted at outpatient clinics of the obstetric and gynecological department at Mansoura University Hospital, Dakahlia Governorate, Egypt.

**Tools of collection:**

Three tools were used for data collection, first tool: Structured Interviewing Questionnaire to collect data about the participant's socio-demographic data and obstetric and infant history. Second tool: Yoga exercise schedule card. This tool functioned as both a guidance and monitoring resource for the postpartum Yoga exercise. Third tool: Quality of Life Questionnaire. This standardized tool was used to assess the overall quality of life of postpartum women.

**Results:**

The results of this study demonstrate a statistically significant difference in postpartum quality of life between the study and control groups (*p* < 0.001). All participants in the study group reported satisfaction across various levels, while none of the control group participants expressed satisfaction.

**Conclusion:**

The study concluded that home-based yoga exercises had a positive effect on improving the quality of life among postpartum women. Respondents who regularly practiced yoga presented a better physical and emotional well-being in comparison to others in the control group.

**Recommendations:**

Postpartum yoga shall be merged into routine maternal healthcare to enhance their physical and emotional well-being after delivery. Accessible, home-based yoga programs with clear instructions to accommodate diverse postpartum conditions shall be developed by the Healthcare authorities. Also, training nurses and midwives on safe yoga practices to lead women and improve the effectiveness, and ensure their safety during performing this intervention.

**Supplementary Information:**

The online version contains supplementary material available at 10.1186/s12978-026-02357-2.

## Introduction

The postpartum period is a crucial phase in women's lives known with deep physical, emotional, and psychological changes. During this time, many face challenges such as fatigue, exhaustion, sleep disturbances, musculoskeletal pain, anxiety, and even depression, which can significantly impair their quality of life (QoL) [1]. Increasingly, non-pharmacological measures are being suggested to reinforce the postpartum recovery, and one of them is yoga, which has gained more attention because of its holistic benefits.

Yoga is an old practice which employees both mind and body, and is commonly familiar with its ability to improve both physical and psychological well-being. Recent studies postulated that postpartum yoga effectively reduces degrees of stress, improves total sleep quality, enhances muscular strength, and thus contributes to emotional stability [2, 3]. Dissimilar, traditional programs such as home-based yoga exercises make new mothers find flexibility to practice at their convenience, which improves their adherence and outcomes, specifically in either the resource-limited or the culturally conservative settings.

As well, yoga exercise gathers physical postures, calm, relaxing breathing techniques, and mindfulness, which have been shown to enhance physical health, promote relaxation, and reduce levels of stress. Moreover, home-based yoga programs deliver practical, cost-effective solutions for postpartum women who may have had childcare responsibilities, limited mobility, or difficulties accessing health services [4].

Additionally, Rahman & El-Gendy [5] in India proved that home-based yoga programs provide women with flexibility and more consistency in practice, empowering them to be engaged in self-care, and have positive impacts of structured home-based yoga programs on postpartum quality of life, including additional improvements in their physical functions, mental health, and overall well-being. These benefits emphasize the value of merging yoga exercises into all the postpartum care approaches. Other studies have revealed that those who engaged in home-based yoga programs reported notable developments in the domains of quality of life as well as emotional regulations, total sleep qualities, and physical strength, given its safety, affordability, and holistic impact, home-based yoga emerges as a promising intervention to support postpartum recovery and enhance maternal well-being [6–8].

Considering quality of life, yoga is recognized as a powerful non-pharmacological intervention, especially during postpartum periods. A study by Ahmed and Soliman [9] declared that postpartum women who regularly exercised yoga for a complete six weeks described a significant reduction in physical discomforts, including fatigue and lower back pain, and also improved mobility. These improvements played a great role in women's ability to perform daily responsibilities and provide care for their babies.

Beyond physical outcomes, yoga positively contributes to both mental and emotional well-being, which act as central dimensions of postpartum quality of life. As mentioned by Kumar and Fathy [10], home-based yoga programs lead to marked reductions in postpartum depressive and stressful symptoms, along with improved levels of emotional regulation and self-efficacy. These results are attributed to the relaxing effects of deep breathing exercises and mindfulness practices combined in yoga. Besides, Nasser and El-Toukhy [11] highlighted the significance of culturally sensitive yoga programs that permit women to engage in wellbeing activities without the burden of social stigma or transportation, thereby enhancing their adherence and long-term engagement.

## Significance of the study

The postpartum period denotes a serious period in a woman’s life, where physical, psychological, and emotional health are significantly affected. In Egypt, many postpartum women experience barriers to receiving comprehensive, extensive postnatal care, mainly in underserved and rural areas. Inadequate accessibility of mental health facilities, cultural norms, and household responsibilities often prevents new mothers from seeking external support, which leads to maleficent postpartum symptoms and diminished quality of life [12].

In Egypt, postpartum women generally face challenges, for example, inadequate access to follow-up care, mood disturbances, fatigue, and a lack of culturally fitting non-pharmacological provisions. While recognition of maternal mental and physical health has improved, practical measures that can be performed at home are still uncommon. An Egyptian study by Youssef and Nabil [13] emphasized that more than 60% of their postpartum women in rural areas had unmet needs related to physical recovery and emotional support. Offering home-based yoga exercises facilitates feasible and cost-effective strategies that can be applied individually, along with the Egyptian Ministry of Health’s recommendations to endorse community-based maternal health [14].

Furthermore, El-Masry and Abdelrahman [15] in Egypt verified that combining mind–body practices like yoga into postpartum care significantly reduced depressive symptoms, improved women's self-efficacy, and enhanced their overall well-being. This study is therefore appropriate and applicable, suggesting a sustainable model that improves the postpartum quality of life and reduces pressure on the Egyptian primary healthcare settings.

This study has great significance as it explores home-based yoga exercises as a culturally accepted, low-cost, and non-pharmacological measure that permits Egyptian postpartum women to help in their recovery techniques within their homes. Yoga also encourages holistic healing by merging physical movements, calming breath, and mindfulness, which can lessen common postpartum problems such as musculoskeletal discomfort, fatigue, anxiety, depression, and sleep disturbances [16].

By focusing on an exercise that doesn’t need progressive resources or recurrent clinical visits, this study supports easily accessible and sustainable maternal and postpartum healthcare approaches in Egypt. The results may inform local maternal healthcare initiatives by providing evidence-based policies mainly for midwives and community nurses on the probable benefits of merging yoga-based education and training into postnatal facilities.

In the context of Egypt's ongoing efforts to improve maternal and child health outcomes, especially under national programs for women's health empowerment, this study offers a timely and practical solution to enhance postpartum quality of life and reduce the burden on healthcare systems [17].

### Study design

This study will adopt a quasi-experimental design with two groups (study and control) to assess the effectiveness of a home-based yoga exercise program on the quality of life among postpartum women.

### Study setting

This study was conducted at the outpatient clinics of the obstetric and gynecological department at Mansoura University Hospital, Dakahlia Governorate, Egypt. The hospital is a governmental healthcare institution that provides a wide range of services, including obstetric, gynecological, and postnatal care to women in Dakahlia Governorate and surrounding areas.

#### Sampling

A purposive sampling technique was used to select eligible postpartum women who attended Mansoura University Hospital for postnatal follow-up during the study period.

The total sample consisted of 300 women, who were divided equally into two groups:

- Study group: Received the home-based Yoga exercise in addition to routine postpartum care.
- Control group: Received only routine postpartum care without any added intervention.

## Sample size

A sample size of 300 participants was targeted. This size is determined based on the sample size, which was calculated using Cochran’s formula at a 95% confidence level, a 5% margin of error, and an estimated population proportion of 0.5.

### Inclusion criteria

Postpartum women who had undergone either normal vaginal delivery or cesarean section and were aged between 18 and 40 years were eligible to participate in the study. Eligible participants were required to have healthy infants without any medical problems and to be free from chronic diseases, previous or current mental disorders, and physical disabilities. In addition, participants had completed at least six weeks postpartum and had obtained medical clearance from their healthcare provider to safely engage in physical activity. Women who underwent cesarean section were included only if they had no postoperative complications such as wound infection, severe pain, or delayed healing. Furthermore, all participants were able to read and understand Arabic instructions and were willing to participate in the study by providing informed consent.

### Exclusion criteria

Women were excluded from the study if they experienced high-risk postpartum complications, including postpartum hemorrhage, severe anemia, or uncontrolled hypertension. In addition, women diagnosed with psychiatric disorders or musculoskeletal conditions that could interfere with safe participation in physical activity were excluded. Participants who were already enrolled in any other postpartum exercise program were also excluded from the study.

### Intervention

Firstly, identifying the postpartum women who are eligible to meet the inclusion criteria. After obtaining informed consent, those in the intervention group were scheduled for initial meetings. During these sessions, a professor from the Faculty of Physical Education directed a face-to-face orientation session with each participant. The meeting was intended to explain the study objectives, draw the outline of the benefits of yoga in the postpartum period, and explain the home-based yoga exercise technique.

The professor cooperated with two female fitness trainers, taking the participants to the fitness center, where he personally guided them through the initial yoga sessions. This session was free to all participants. In this practical setting, we directly provided hands-on training to ensure that each woman performs the proper movements. These sessions focused on posture alignment, calm breathing techniques, and safe transitions between positions. This direct supervision helped participants gain confidence, ensure they mastered the basics before continuing the exercises, learned the correct techniques for breathing, aligned postures, and pelvic floor engagement. They were encouraged to interact and ask questions to ensure full understanding of the techniques.

To support home-based exercises, each participant woman was given a video guide that declared the full sequence of yoga exercises in a step-by-step, clear manner. The videos were designed to be easily followed and compatible with smartphones, enabling the women to conveniently access them at home. Each video involved modified postures suitable for different postpartum recovery levels, with emphasis on gradual progression and safety.

Participant women were instructed to apply the yoga exercises at their homes for three to five times per week for eight weeks, each session lasts for approximately 20–30 min. Also, they were provided with a printed log sheet to document their daily exercise, which assisted in tracking their commitment and adherence**.**

#### Key exercises included

- Diaphragmatic (deep belly) breathing for stress reduction and relaxation.
- Pelvic floor (Kegel) exercises to restore muscle tone.
- Cat-Cow stretches to increase spinal flexibility and alleviate back pain.
- Bridge pose to strengthen the lower back and pelvic area.
- Child’s pose and seated forward bends to gently stretch and relax muscles.
- Legs up the wall pose for circulation and calming effects.

Phone calls or WhatsApp messages were used to conduct weekly follow-ups by the research team to address any concerns, provide motivation, and ensure commitment and adherence to the program. They discussed any challenges faced by the participant women and guided them to adapt or modify the exercises if needed.

This supportive, structured approach aimed to develop participants’ engagement and ensure their safe and correct implementations of the home-based yoga exercises, thus leading to brilliant improvements in postpartum quality of life.

## Data collection

Data collection for this study lasted for six months (from the first of July 2024 till the end of December, 2024)**.** Three tools were utilized for data collection:

- Tool I: structured interviewing questionnaire

This tool was developed by the researchers and included two main parts:

- Part 1: Sociodemographic data

This part was used to collect basic demographic information about postpartum women, including ages, places of residence, levels of education, telephone numbers, occupations, and their incomes.

- Part 2: Obstetric and Infant History

This part was used to collect comprehensive data concerning the women’s previous obstetric history, including the number of gravidities, number of parities, type of delivery, their antenatal and postnatal history, and the duration since previous delivery. Additionally, information related to the infants was collected, such as gender, feeding method, type of daily care (e.g., home with mother, nursery, or babysitter), and the infant’s health status.

Data was collected using a self-administered questionnaire, allowing the participant women to freely report their sociodemographic and clinical background.

- Tool II: yoga exercise schedule card

This tool was used as a resource of guidance and monitoring for the postpartum Yoga exercises. The Yoga exercise’s Schedule Card comprised the evidence-based guidelines for safe practices of yoga during the postpartum period, in accordance with many recommendations from the American Pregnancy Association.

This card was delivered to postpartum women with clear, easy instructions on the safe performance of each yoga pose, as well as posture alignment, breathing techniques, and relaxation methods. It also emphasized the physical and psychological benefits of yoga, such as improved flexibility, pelvic floor strength, stress reduction, and emotional well-being (Field, 2021; Kinser & Jallo, 2020).

Besides being a guide, this card functioned to track the participants' adherence to the yoga regimen. It involved structured sections where each participant could record the following:

- Date and duration of each home-based yoga session.
- Type of exercises performed (e.g., stretching, breathing, relaxation).
- Number of repetitions and time spent per exercise.

This tracking mechanism enabled the researchers to monitor the frequency, consistency, and engagement of each woman throughout the 8-week intervention, which was essential for evaluating the impact of yoga on quality of life.

- Tool III: quality of life questionnaire

This was a standardized tool used to evaluate the overall quality of life of postpartum women. Originally, it was developed by Pamela Dee Hill (2018) and adapted from the work of Ware & Sherbourne (1992). This questionnaire assesses satisfaction across various domains of life (e.g., physical health, social functioning, emotional well-being). “The internal consistency of the Quality of Life Questionnaire was assessed using Cronbach’s alpha coefficient. The scale demonstrated good reliability in the current study (α = 0.87).”

- Scoring System:
  - Each subscale was scored independently, and responses were converted into a 0–100 scale, with higher scores indicating better quality of life.

This instrument offered a reliable questionnaire on the impact of Yoga exercise on the postnatal women's well-being.

### Data collection procedures

The data collection procedures involved three main phases: preparation, implementation, and evaluation.

- 1. Preparation phase

In this phase, the researcher developed the needed tools for data collection: the structured self-administered questionnaire (Tool I), the Yoga exercise Schedule Card (Tool II), and the standardized Quality of Life Questionnaire (Tool III). The developed tools were checked by a panel of experts in maternal health nursing and physical education to ensure their content validity and appropriateness for the targeted population. Ethical approvals were obtained, and permissions were secured from the selected healthcare setting.

Training sessions were organized for the research team, in collaboration with a professor from the Faculty of Physical Education, who oversaw the yoga orientation and gym activities. To further support participants, a video tutorial was created, offering clear, step-by-step guidance for practicing yoga exercises at home.

- 2. Implementation phase

Postpartum women who met the inclusion criteria within the selected healthcare setting were recruited. Following a clear explanation of the study objectives and the acquisition of verbal informed consent, participants were enrolled and allocated to either the intervention group or the control group.

All participants completed the baseline data collection using Tool I, which covered sociodemographic details and obstetric and infant history.

Participant women assigned to the intervention group were invited to participate in a free yoga training session at the gym, conducted by the professor of physical education. In this face-to-face session, the professor demonstrated the yoga postures, pelvic floor engagement, breathing techniques, and relaxation methods. Women were encouraged to engage actively by asking questions and practicing the movements under his supervision. To support the continuity of practice at home, each participant received a Yoga exercise Schedule Card and a smartphone-compatible instructional video.

Over 8 weeks, the intervention group followed the yoga protocol at home, using the schedule card to record:

- The date and duration of each session,
- Types of exercises performed (e.g., stretching, breathing, relaxation),
- Repetitions and time spent per exercise.

The researcher maintained regular contact and visited the healthcare setting three times per week to provide support, answer questions, and ensure adherence to the study protocol.

- 3. Evaluation phase

At the end of the 8-week intervention period, both groups completed the Quality-of-Life Questionnaire (Tool III).

## Timeline

The study was conducted over six months, with the first month dedicated to preparatory activities, including content development and participant recruitment. The intervention and data collection will occur in the following two months, and the final three months will be allocated to data analysis and reporting.

## Ethical considerations

### Informed consent

Oral informed consent was obtained from participants.

### Confidentiality

Participants' identities were kept confidential, and data will be anonymized.

### Ethical approval

The study protocol was reviewed and approved by the Institutional Review Board (IRB)of the Faculty of Physical Education, Mansoura University.

### Pilot study

A pilot study was carried out on 30 women to test the objectivity and applicability of the research tools and the feasibility of the research process. Participants in the pilot study will be excluded from the research study. The pilot study revealed the feasibility, effectiveness, and appropriateness of the study instruments.

### Statistical analysis of the data

The statistical analysis of the data was performed using IBM SPSS software version 20.0(Armonk, NY: IBM Corp, released 2011). Categorical data were summarized as numbers and percentages. To compare the two studied groups, the Chi-square test was used. Normality was assessed using the Kolmogorov–Smirnov test. Monte Carlo correction for chi-square when more than 20% of the cells have expected count less than 5. Quantitative data were presented as range, mean, and standard deviation. Student t-test for normally distributed quantitative variables, to compare between two studied groups. F-test for normally distributed quantitative variables, to compare more than two groups.

## Results

As shown in Table 1, the study revealed significant differences between the study and control groups in several key characteristics. Notably, age distribution varied significantly, with the majority of the study group aged 25–29 years, while the control group had more participants in the 30–35 age range. Educational level also showed a highly significant difference, as most women in the study group had university-level education, compared to a higher proportion of secondary-level education in the control group. Regarding occupation, a greater number of housewives were observed in the study group, whereas more women in the control group were employed, which may influence the time available for engaging in the intervention.

**Table 1:** Comparison between the two studied groups according to demographic data

Demographic data	Study (*n* = 150)	Control (*n* = 150)	χ^2^	P
Age (years)				
18–24 Years	41 (27.3%)	30 (20.0%)	28.210^*^	< 0.001^*^
25–29 Years	68 (45.3%)	36 (24.0%)		
30–35 Years	29 (19.3%)	69 (46.0%)		
36–40 Years	12 (8.0%)	15 (10.0%)		
Educational level				
Primary level	4 (2.7%)	0 (0.0%)	29.741^*^	^MC^p < 0.001^*^
Secondary level	27 (18.0%)	69 (46.0%)		
University level	119 (79.3%)	81 (54.0%)		
Occupation				
House wife	130 (86.7%)	100 (66.7%)	16.770^*^	< 0.001^*^
Working	20 (13.3%)	50 (33.3%)		
Place of residence				
Urban	130 (86.7%)	117 (78.0%)	3.873^*^	0.049^*^
Rural	20 (13.3%)	33 (22.0%)		
Monthly Income for an individual				
Not enough	10 (6.7%)	11 (7.3%)	2.737	0.255
Enough	91 (60.7%)	77 (51.3%)		
Enough and save	49 (32.7%)	62 (41.3%)		
BMI(kg/m2)				
Over weight	129 (86.0%)	121 (80.7%)	5.589	^MC^p = 0.061
Normal weight	19 (12.7%)	19 (12.7%)		
Under weight	2 (1.3%)	10 (6.7%)		
Do you have any problems during the postpartum period?				
No	150 (100.0%)	150 (100.0%)	–	–
Yes	(0.0%)	(0.0%)		
Number of living children				
One	44 (29.3%)	11(7.3%)	79.534^*^	^MC^p < 0.001^*^
Two	65 (43.3%)	24 (16.0%)		
Three	41 (27.3%)	106 (70.7%)		
Four or more	0 (0.0%)	9 (6.0%)		

χ^2^: Chi-square test, MC: Monte Carlo test

p: *p*-value for comparing between the two studied groups*: Statistically significant at *p* ≤ 0.05

Additionally, a significant difference was found in the number of living children, with most study group participants having fewer children, potentially allowing more flexibility in practicing yoga at home. Although differences in place of residence were marginally significant, both monthly income and BMI were not statistically different between the groups, indicating reasonable homogeneity in these areas. All participants reported no postpartum complications.

Refer to Table 2, the study indicated statistically significant differences between the study and control groups in both types of labor and type of feeding. Regarding labor outcomes, the study group had a notably higher rate of emergency cesarean Sects. (14.0%) compared to none in the control group, while elective cesarean sections were more prevalent among controls (82.0% vs. 63.3%). Interestingly, spontaneous vaginal delivery was less frequent in the study group (3.3%) compared to the control group (9.3%), suggesting variations in labor progression or medical decision-making. These differences were statistically significant (*p* < 0.001), reflecting the potential influence of the intervention or other clinical factors on delivery mode.

**Table 2:** Comparison between the two studied groups according to Delivery outcome

Delivery outcome	Study (*n* = 150)	Control (*n* = 150)	χ^2^	P
Type of labor				
Normal spontaneous Vaginal delivery	5 (3.3%)	14 (9.3%)	34.955^*^	< 0.001^*^
Normal delivery with Episiotomy	29 (19.3%)	13 (8.7%)		
Elective Cesarean Section	95 (63.3%)	123 (82.0%)		
Emergency Cesarean Section	21 (14.0%)	0 (0.0%)		
Post – date delivered	0 (0.0)	0 (0.0%)		
Gestational Age				
> 37	28 (18.7%)	40 (26.7%)	2.738	0.098
37 – 40	122 (81.3%)	110 (73.3%)		
Type of feeding				
Breastfeeding	118 (78.7%)	96 (64.0%)	13.551^*^	0.001^*^
Bottle feeding	13 (8.7%)	9 (6.0%)		
Bottle and breastfeeding	19 (12.7%)	45 (30.0%)		

χ2: Chi-square test

p: *p*-value for comparing between the two studied groups

^*^: Statistically significant at *p* ≤ 0.05

As for feeding practices, the majority of women in the study group practiced exclusive breastfeeding (78.7%), significantly higher than the control group (64.0%). In contrast, mixed feeding (bottle and breastfeeding) was more common among control participants (30.0% vs. 12.7%). This variation was also statistically significant (*p* = 0.001), which may reflect increased awareness, support, or confidence in breastfeeding among the study group. No significant difference was observed in gestational age distribution between the two groups (*p* = 0.098), indicating comparability in timing of delivery.

Refers to Table 3 illustrates a clear difference between the study and control groups regarding quality-of-life levels. None of the women in the study group reported being dissatisfied, while the majority of the control group fell within dissatisfied categories, with more than half (57.3%) being very dissatisfied and 40% moderately dissatisfied. In contrast, the study group showed notable improvement, as more than half (51.3%) were slightly satisfied, over one-third (36.7%) moderately satisfied, and 12% reported being very satisfied.

**Table 3:** Comparison between the two studied groups according to yoga score

Quality of life	Study (*n* = 150)	Control (*n* = 150)	Test of Sig	P
**Level**				
Dissatisfied				
Very Dissatisfied (0% – 16.6%)	0 (0.0%)	86 (57.3%)	ꭓ^2^ = 300.000^*^	< 0.001^*^
Moderately Dissatisfied (16.7% – 33.2%)	0 (0.0%)	60 (40.0%)		
Slightly Dissatisfied (33.3% – 49.8%)	0 (0.0%)	4 (2.7%)		
Satisfied				
Slightly Satisfied (49.9% – 66.4%)	77 (51.3%)	0 (0.0%)		
Moderately Satisfied (66.5% – 83.0%)	55 (36.7%)	0 (0.0%)		
Very Satisfied (83.1% – 100%)	18 (12.0%)	0 (0.0%)		
Total score (27 – 162)				
Min. – Max	108.0 – 162.0	27.0 – 73.0	t = 45.023^*^	< 0.001^*^
Mean ± SD	122.9 ± 14.93	45.63 ± 14.81		
**Average score (1–6) (Mean ± SD.)**	4.55 ± 0.55	1.69 ± 0.55		
**Percent score (Mean ± SD)**	71.07 ± 11.06	13.80 ± 10.97		

*SD* Standard deviation, *t* Student t-test, χ^2^ Chi-square test

p: *p*-value for comparing between the two studied groups

^*^: Statistically significant at *p* ≤ 0.05

The comparison of mean scores further highlights this disparity, where the study group achieved a significantly higher total score (122.9 ± 14.93) compared to the control group (45.63 ± 14.81), with a highly significant statistical difference (*p *< 0.001). The percent score and average satisfaction levels also confirm this finding, reflecting an overall higher quality of life among women in the study group compared to those in the control group.

As presented in Table 4, the study illustrated the engagement level of postpartum women in the study group regarding the frequency and duration of yoga sessions. The majority of participants (69.3%) reported practicing yoga for 20 min per session, indicating that this duration was the most practical and acceptable among the group. Only a small proportion practiced for either 10 min (26.7%) or 30 min (4.0%), suggesting that extremely short or longer sessions were less favored.

**Table 4:** Distribution of the studied cases according to different parameters in the study group (*n* = 150)

	No. (%)
Duration of yoga exercises that postpartum women do in every session (minutes)	
10	40 (26.7)
20	104 (69.3)
30	6 (4.0)
Number of Sessions per Week	
3	8 (5.3)
4	90(60.0)
5	52 (34.7)

In terms of weekly frequency, most women performed yoga four times per week (60.0%), followed by five times per week (34.7%). A minimal number of participants (5.3%) practiced only three times weekly. These findings reflect a generally high level of adherence to the yoga program, with most participants engaging consistently throughout the week. This level of commitment could be associated with the accessibility and simplicity of the home-based intervention, as well as the perceived benefits experienced by the mothers.

As refer to Table 5 demonstrates a statistically significant relationship between the type of delivery and the total quality of life scores among the study group (*p* < 0.001). Women who underwent elective cesarean section reported the highest mean score (126.88 ± 16.72), while those who had normal spontaneous vaginal delivery recorded the lowest (110.0 ± 1.41), followed by emergency cesarean Sect. (112.57 ± 2.46). Mothers who delivered vaginally with episiotomy achieved an intermediate mean score (119.76 ± 8.34). This indicates that the mode of delivery may have a considerable impact on perceived quality of life, with elective cesarean section being associated with higher satisfaction levels compared to other modes.

**Table 5:** Relation between total score of Quality of life with Delivery outcome in study group (*n* = 150)

	**N**	**Total score of Quality of life**	**Test of Sig**	**p**
		**Mean ± SD**		
Type of labor				
Normal spontaneous Vaginal delivery	**5**	110.0 ± 1.41	F = 8.355^*^	< 0.001^*^
Normal delivery with Episiotomy	**29**	119.76 ± 8.34		
Elective Cesarean Section	**95**	126.88 ± 16.72		
Emergency Cesarean Section	**21**	112.57 ± 2.46		
Post – date delivered	**0**	–		
Gestational Age				
> 37	**28**	111.46 ± 1.26	t = 4.837	< 0.001^*^
37 – 40	**122**	125.57 ± 15.38		
Type of feeding				
Breastfeeding	**118**	125.36 ± 15.59	F = 7.993^*^	0.001^*^
Bottle feeding	**13**	114.54 ± 1.51		
Bottle and breastfeeding	**19**	113.63 ± 9.07		

SD: Standard deviation F: F for One-way ANOVA testt: Student t-test

p: p-value for comparing between different categories

^*^: Statistically significant at *p* ≤ 0.05

Gestational age at delivery also showed a significant effect. Women who delivered at term (37–40 weeks) had a higher mean score (125.57 ± 15.38) compared to those who delivered after 37 weeks only (> 37 weeks) (111.46 ± 1.26), with a statistically significant difference (*p* < 0.001).

Regarding feeding practices, the results revealed significant differences as well (*p* = 0.001). Mothers who practiced breastfeeding recorded the highest mean score (125.36 ± 15.59), whereas those who relied on bottle feeding (114.54 ± 1.51) or mixed feeding (113.63 ± 9.07) reported lower levels of quality of life. This highlights the positive influence of breastfeeding on enhancing postpartum quality of life compared to artificial or mixed feeding methods.

## Discussion

The transition to motherhood is a significant life event that can affect well-being in many ways. The postpartum period brings new challenges that can lower quality of life (QoL). Women may experience physical discomfort, decreased socialization, and increased responsibilities at home. Motherhood can lead to greater satisfaction but may also create stress [18]. Postpartum life can be a volatile emotional phase, prompting the need for greater emotional control, satisfaction, companionship, and health awareness. However, the time demand of childcare often prevents attention to the women themselves. Researchers have focused on yoga as a potential support during this transition. Yoga incorporates goals that align well with common postpartum psychological and physical issues [19].

Despite the potential benefits of yoga in the postpartum period, finding an accessible format remains a challenge. Although studio classes are the most common delivery format, obstacles such as time constraints, travel distances, and costs can hinder participation. Researchers investigated the impact of a home-based program that does not require instructor interaction. These programs are easier to promote and monitor. A quasi-experimental study by Nadholta et al. [20] was designed to evaluate the impact of 16 sessions of home-based yoga on postpartum QoL in a group setting in which instructor presence is unnecessary. Participants were encouraged to practice independently over 8 weeks, performing 2 sessions per week, but they were free to adjust attendance according to their schedules.

In our study, the demographic characteristics of the study and control groups showed statistically significant differences in variables such as age, educational level, occupation, place of residence, and number of living children. These differences may have influenced participants' engagement with the intervention and outcomes. The evaluation indicates that the home-based yoga intervention did not yield statistically significant improvements in any quality-of-life domain relative to the control participants following an 8-week period. Adjusted analyses controlling for baseline covariates, however, showed substantially sized effects on the mental health and environmental domains coupled with small improvements in the physical and social domains and a clinically relevant increase in overall quality of life. Prior meta-analytic work indicates that effect sizes of at least this magnitude among postpartum individuals generally signify a clinically meaningful change [21]. Accordingly, while the absence of statistically significant group contrasts suggests that the intervention cannot be considered effective in the normative sense, the magnitude of the observed changes warrants further consideration.

Research into quality of life after childbirth is scarce, particularly with regard to quasi-experimental and non-intervention studies. The few pre-post studies on the effects of prenatal/postnatal exercise interventions indicate increases in quality of life, yet variability of the physical activities included and concurrent improvement in general health prevent clear attribution to exercise itself [22]. In a larger cohort, parous participants in a randomized controlled trial of stand-alone, mid-exercise, and post-exercise yoga reported greater heightened quality of life than null—complementing general health and mental well-being yet diverging from health-related quality of life—for all three conditions when compared with those receiving standard obstetric care. Taken together, these results suggest that yoga renders substantial enhancement to quality-of-life experiences among postpartum individuals [23].

On testing, the internal validity was notably compromised by the small sample size and corresponding low statistical power. Neither randomization nor matching for confounding variables could be implemented. The possibility of systematically differing baseline covariates between the two groups therefore remains, as does the lack of formal blinding for nearly all contacts throughout the study. Given that both the participants in the experimental condition and the investigator were aware of group allocation, post-intervention differences remain ambiguous with respect to causality. The adoption of an a priori, individual-level, intention-to-treat framing required substantially less pruning of the dataset, allowed retention of initial sample size and balance of pre-intervention covariates, and aligned with standard academic convention. The substantial dropout and consequent risk of bias therefore arise from non-adherence to the intervention rather than from ethical or similar considerations; the question of adequate data pertained solely to whether the retained set permitted within-group analysis.

Similar to Xing, [24], conception of the yoga program without direct application to babies limited the class components to the postpartum period,feedback indicated frequent consideration of anticipated return to caregiving duties. The tendency for interventions targeting the mother individually to elicit participant preference yet for the shared parent-infant focus to motivate attendance remains evident throughout, potentiating avoidance of perceived group limitations. Ubiquity of non-parental external influence during caregiving omits the progression of shared practice opportunities among participants and their children. Whereas demand for a broad physical and educational agenda initially seemed well-documented, securing sustaining locations and personnel proved considerably more challenging than anticipated. Proposed adaptations remain viable under similar conditions.

Additionally, home-based yoga showed promise for enhancing quality of life in postpartum women. The study’s findings align with other evidence indicating home-based yoga can improve outcomes during the perinatal period. Similarly, a systematic review by Munns et al., [21] noted the beneficial effects of yoga on postnatal mental health and well-being, encompassing anxiety, stress, mood, depressive symptoms, self-compassion, and quality of life. Interventions varied widely in design, delivery format, duration, and participant characteristics, making direct comparisons difficult. Factors such as support from family, friends, and health professionals as well as the convenience of video tutorials were identified as facilitators or barriers to attendance.

Moreover, a randomized trial by Carlos Sanchez-Garcia et al., [22] examined hypo-pressive exercise, a physical fitness program regulated by breathing and performed in various postures, among postpartum women. Participants in an experimental group completed hypo-pressive sessions twice weekly at home for 30 min over 12 weeks. Their results indicated a significant increase in health-related quality of life in the experimental group compared with the control group, consistent with previous research demonstrating that supervised or unsupervised exercise during pregnancy or after childbirth positively influences quality of life.

These observations suggest that comparable gains might be expected among postpartum women who practice home-based yoga compared to similar women who do not engage in regular physical activity. The only available quasi-experimental study of home-based yoga in postpartum populations similarly favored intervention participants in practices involving regular physical activity, underscoring a widespread consensus regarding the benefits of physical activity during the fourth trimester.

Existing quasi-experimental evidence indicates mixed effects of yoga on postpartum quality of life. An uncontrolled study of online yoga for perinatal women reported improved life satisfaction, mastery, and psychological acceptance but not emotional well-being, physical health, or motherhood contentment [21]. In a UK-based evaluation of the Mum & Baby Yoga online intervention, participants showed significant reductions in depression, anxiety, insomnia, and stress, accompanied by improvement in the quality of mother–child interactions. Two Australian and Indian studies of yoga combined with peer support and education indicated benefits for social engagement and community connectedness, but no significant improvements in depression, anxiety, or sleep problems were observed 25, 26.

In their study, Nilsson, et al., [27] reported average adherence to the home-based yoga intervention (i.e., 4.5 sessions per week) exceeds adherence to online intervention generally (two sessions across a 6-week period, with six videos). The combination of online delivery and home-based symmetry with social gatherings, exercises, music, and sleep meditation resonates with characteristics of other interventions received positively in postpartum populations. This quasi-experimental study assessed the effects of home-based yoga practice on quality of life in postpartum women and compared the objective findings with existing quasi-experimental evidence.

In this study, quality-of-life indices improved in the intervention group and mood, physical recovery, and sleep need showed favorable changes. These results indicate that home-based yoga practice benefits postpartum women, corroborating previous reports, and facilitate evidence-based recommendations to health-care providers and society. Quality of life during the postpartum period varied considerably among women and was lower than population norms. The quality-of-life indices reported after the home-based yoga intervention fell within the classification of mild quality-of-life deficiency suggested by the population database, although the necessity of restoring quality of life to pre-pregnancy status had not been explicitly established.

Adjunctive home practice in the study by Munns et al., [21] constitute an essential component of modern yoga instruction, yet adherence to a home program remains a challenge for practitioners in general, not only postpartum individuals. Increased frequency of home practice correlates with more substantial subjective benefit from yoga. Postpartum women engaged in group-home practice spontaneously moderated the intensity of practice, which still resulted in significant benefits. Although several sessions provided direct experience of the methods to promote higher engagement, further investigation in controlled studies of the impact of practice moderation on subjectively perceived benefits and adaptations ‘including gradual decontraction of physical repertoires or reduction of all practices’ seems warranted.

Postpartum yoga is generally considered safe when initiated after an appropriate recovery period (typically after 6 weeks postpartum for both vaginal and cesarean deliveries), and following medical approval.Clinically, postpartum yoga has been shown to:Improve pelvic floor and abdominal muscle strength,Reduce lower back pain and physical discomfort,Enhance psychological well-being by reducing anxiety and depressive symptoms, and Promote circulation and overall physical recovery.

### limitations of the study

This study has several limitations that should be considered when interpreting the findings. First, the use of purposive sampling may limit the generalizability of the results to the wider population of postpartum women. In addition, the study was conducted at a single center, which may reduce the external validity of the findings. The study also relied primarily on self-reported data, which may have introduced reporting or recall bias. Furthermore, the relatively short follow-up period limited the ability to assess the long-term effects of home-based yoga exercises on postpartum women’s quality of life. Finally, the absence of objective clinical outcome measures may have restricted the comprehensive evaluation of the intervention’s effectiveness.

## Conclusions

Effective home-based yoga exercises significantly improved quality of life indicators across multiple domains (including social support and fatigue) in postpartum women, alongside secondary enhancements in mood, physical recovery, and sleep. Women receiving yoga at home enjoyed high adherence and found the intervention acceptable, endorsing ongoing yoga practice. Yet comparisons with existing quasi-experimental quasi-experimental evidence confirmed improvements in postpartum quality of life are achievable irrespective of intervention type. In fact, the impact of home-based yoga practice remains uncertain in this setting.

Quasi-experimental intervention designs support causal assertions while facilitating studies that would otherwise be impossible or unethical. However, the absence of random assignment constrains the generalizability of quasi-experimental conclusions, underscoring the need for objective situating of findings within existing quasi-experimental evidence.

### Recommendation

Based on these findings, it is recommended that postpartum yoga be incorporated into standard maternal care to enhance women’s physical and emotional recovery after childbirth. Enhanced community and institutional support for this relatively low-cost intervention is warranted, given evidence suggesting substantial yoga benefits for postpartum wellness. In addition, the implementation options include postpartum yoga resources promoted through mass media or social networks, partnerships with community on maternal health, and yoga-specific organizational programs that could encompass various home-based activities.

## Supplementary Information

Supplementary Material 1.

Supplementary Material 2.

Supplementary Material 3.

Supplementary Material 4.

## Data Availability

No datasets were generated or analysed during the current study.
