# Supplementary material for: The effect of home-based yoga exercises on quality of life among postpartum women
Source: Reprod Health. 2026 Jun 13;23:114. doi: 10.1186/s12978-026-02357-2 (PMC13264819; doi:10.1186/s12978-026-02357-2)
Supplement: Supplementary file 1 — Supplementary Material 1. [file 12978_2026_2357_MOESM1_ESM.docx]

**Reviewer Comment:**
For inclusion, exclusion, and limitations please draft the sections as a narrative rather than as bullet points.

**Response:**
Thank you for this valuable comment. The Inclusion Criteria, Exclusion Criteria, and Limitations of the Study sections have been revised and rewritten in a narrative format instead of bullet points, as requested. These modifications were made to improve the readability and consistency of the manuscript according to the journal guidelines.

**Changes Made in the Manuscript:**

- The Inclusion Criteria section was rewritten into a descriptive narrative paragraph.
- The Exclusion Criteria section was revised into a narrative format.
- The Limitations of the Study section was also rewritten as a narrative paragraph with clearer explanation of the study limitations.
