# Supplementary material for: The effect of home-based yoga exercises on quality of life among postpartum women
Source: Reprod Health. 2026 Jun 13;23:114. doi: 10.1186/s12978-026-02357-2 (PMC13264819; doi:10.1186/s12978-026-02357-2)
Supplement: Supplementary file 4 — Supplementary Material 4. [file 12978_2026_2357_MOESM4_ESM.docx]

**Study Design:**

This study will adopt a **quasi-experimental design with two groups (study and control)** to assess the effectiveness of a home-based yoga exercise program on the quality of life among postpartum women.

**Study Setting**:

This study was conducted at outpatient clinics of the obstetric and gynecological department at Mansoura University Hospital, Dakahlia Governorate , Egypt.. The hospital is a governmental healthcare institution that provides a wide range of services, including obstetric, gynecological, and postnatal care to women in Dakahlia Governorate and surrounding areas.

**Sampling**

A **purposive sampling technique** was used to select eligible postpartum women who attended Mansoura University Hospital for postnatal follow-up during the study period.

The total sample consisted of **(300 )** women, who were divided equally into two groups:

- **Study group**: Received the home-based yoga intervention in addition to routine postpartum care.
- **Control group**: Received only routine postpartum care without any added intervention.

**Sample Size:**

A sample size of 300 participants was be targeted. This size is determined based on The sample size was calculated using Cochran’s formula at a 95% confidence level, a 5% margin of error, and an estimated population proportion of 0.5.

**Inclusion Criteria**

- Postpartum women after normal delivery and cesarean section aged 18 -40 years.
- Women having babies without any health problems, no chronic diseases, no previous or current mental disorders, not having any physical disability,
- Within the **first 6 months postpartum**.
- Medically stable and cleared for light physical activity.
- Able to read and understand instructions in Arabic.
- Willing to participate and provide informed consent.

**Exclusion Criteria:**

• Women with **high-risk postpartum complications** (e.g., hemorrhage, severe anemia, or uncontrolled hypertension).

• Diagnosed **psychiatric or musculoskeletal conditions** that prevent safe physical activity.

• Participation in any other postpartum exercise program.

**Intervention:**

At the beginning of the study identifying eligible postpartum women who met the inclusion criteria. After obtaining informed consent, the women in the intervention group were scheduled for an initial meeting. During this session, a professor from the Faculty of Physical Education conducted **a face-to-face orientation session** with each participant. The purpose of the meeting was to explain the study objectives, outline the benefits of yoga in the postpartum period, and introduce the home-based exercise protocol.

The professor from the Faculty of Physical Education collaborated with two female fitness trainers working accompanied the participants to the gymnasium, where he personally guided them through the initial yoga sessions. This session was offered free of charge to all participants. In this practical setting, he provided hands-on training to ensure each woman correctly performed the movements. The sessions focused on posture alignment, breathing techniques, and safe transitions between poses. This direct supervision helped build participants’ confidence and ensured they mastered the fundamentals before continuing the exercises at ensuring that each participant learned the correct techniques for breathing, posture, pelvic floor engagement, and relaxation. Participants were encouraged to ask questions to ensure full understanding of the routines.

To support home practice, each woman was given a **video guide** that demonstrated the full sequence of yoga exercises in a clear, step-by-step manner. The video was designed to be easy to follow and compatible with smartphones, enabling participants to access it conveniently at home. The video included modified postures suitable for different levels of postpartum recovery, with emphasis on safety and gradual progression.

Participants were instructed to perform the yoga exercises at home three to five times per week for eight weeks, with each session lasting approximately 20–30 minutes. They were also provided with a printed exercise log sheet to record their daily practice, which helped in tracking adherence and commitment**.**

**Key Exercises Included:**

- Diaphragmatic (deep belly) breathing for stress reduction and relaxation.
- Pelvic floor (Kegel) exercises to restore muscle tone.
- Cat-Cow stretches to increase spinal flexibility and alleviate back pain.
- Bridge pose to strengthen the lower back and pelvic area.
- Child’s pose and seated forward bends to gently stretch and relax muscles.
- Legs up the wall pose for circulation and calming effects.

Weekly follow-up was conducted via phone calls or WhatsApp messages by the research team to provide motivation, address any concerns, and ensure compliance with the program. Any challenges faced by the participants were discussed, and guidance was given to adapt the exercises if needed.

This structured and supportive approach aimed to enhance participant engagement and ensure the correct and safe implementation of the yoga exercises, ultimately contributing to the improvement of postpartum quality of life.

**Data collection:**

Data collection for this study was carried out over a six-month period, from the beginning of **July 2024 to the end of December 2024**. The researchers utilized **three tools** to collect the required data:

**Tool I: Structured Interviewing Questionnaire**
This tool was developed by the researchers and composed of two main parts:

- **Part 1: Sociodemographic Data**
  This section collected basic demographic information about postpartum women, including age, level of education, place of residence, telephone number, occupation, and income.
- **Part 2: Obstetric and Infant History**
  This section gathered comprehensive data regarding the women’s obstetric history, including gravidity, parity, antenatal and postnatal history, type of delivery, and time since previous delivery. Additionally, information related to the infant was collected, such as sex, feeding method, type of daycare (e.g., home with mother, babysitter, or nursery), and the infant’s health status.
  The data was collected using a **self-administered questionnaire**, allowing participants to report their sociodemographic and clinical background comfortably.

**Tool II: Yoga Exercise Schedule Card:**

This tool functioned as both a **guidance and monitoring resource** for the postpartum yoga intervention. The **Yoga Exercise Schedule Card** included evidence-based guidelines for the **safe practice of yoga during the postpartum period**, in accordance with recommendations from the **American Pregnancy Association**.

The card provided postpartum women with clear instructions on how to perform each yoga pose safely, including breathing techniques, posture alignment, and relaxation methods. It also highlighted the **physical and psychological benefits** of yoga, such as improved pelvic floor strength, enhanced flexibility, stress reduction, and emotional well-being **(Field, 2021; Kinser & Jallo, 2020).**

In addition to serving as a guide, the card was used to **track the participants' adherence** to the yoga regimen. It contained structured sections where each participant recorded the:

- **Date and duration** of each home-based yoga session.
- **Type of exercises** performed (e.g., stretching, breathing, relaxation).
- **Number of repetitions** and time spent per exercise.

This tracking mechanism enabled the researchers to monitor the **frequency, consistency, and engagement** of each woman throughout the 8-week intervention, which was essential for evaluating the impact of yoga on quality of life.

**Tool III: Quality of Life Questionnaire**
This standardized tool was used to assess the overall quality of life of postpartum women. It was originally developed by **Pamela Dee Hill (2018)** and adapted from the work of **Ware, J.E., & Sherbourne, C.D. (1992)**. The questionnaire evaluates satisfaction across multiple domains of life (e.g., physical health, emotional well-being, social functioning).

- **Scoring System:** Each subscale is scored independently, and responses are converted to a **0–100 scale**, with **higher scores indicating better quality of life**.
  This instrument provided a reliable measurement of the impact of the yoga intervention on women's postnatal well-being.

**Data Collection Procedures:**

The data collection process was implemented in three main phases: **preparation**, **implementation**, and **evaluation**.

**1. Preparation Phase**

During this phase, the researcher developed the necessary tools for data collection, including the structured self-administered questionnaire (Tool I), the Yoga Exercise Schedule Card (Tool II), and the standardized Quality of Life Questionnaire (Tool III). The tools were reviewed by a panel of experts in maternal health, nursing, and physical education to ensure content validity and appropriateness for the target population. Necessary ethical approvals were obtained, and permissions were secured from the selected healthcare setting.

Training was also conducted for the research team, including coordination with the professor from the Faculty of Physical Education, who was responsible for delivering the yoga orientation and gym sessions. Additionally, a video guide for the home-based yoga exercises was developed to provide clear, step-by-step instructions for participants.

**2. Implementation Phase**

Eligible postpartum women were identified based on inclusion criteria from the selected healthcare setting. After explaining the study’s objectives and obtaining informed oral consent, participants were enrolled and assigned to either the intervention or control group.

All participants completed the baseline data collection using **Tool I**, which covered sociodemographic details and obstetric and infant history.

Women in the **intervention group** were invited to attend a **free yoga training session at the gym**, led by the professor of physical education. During this face-to-face session, the professor demonstrated the yoga postures, breathing techniques, pelvic floor engagement, and relaxation methods. Participants were encouraged to ask questions and practice the movements under supervision. Each participant received a **Yoga Exercise Schedule Card** and a smartphone-compatible video to guide their home practice.

Over an 8-week period, the intervention group followed the yoga protocol at home, using the schedule card to record:

- The date and duration of each session,
- Types of exercises performed (e.g., stretching, breathing, relaxation),
- Repetitions and time spent per exercise.

The researcher maintained regular contact and visited the healthcare setting three times per week to provide support, answer questions, and ensure adherence to the study protocol.

**3. Evaluation Phase**

At the end of the 8-week intervention period, both groups completed the **Quality of Life Questionnaire (Tool III)**

**Time line**

The study was conducted over six months, with the first month dedicated to preparatory activities, including content development and participant recruitment. The intervention and data collection will occur in the following two months, and the final three months will be allocated to data analysis and reporting.

**Ethical Considerations**

**Informed Consent:** Oral informed consent was obtained from participants.

**Confidentiality:** Participants' identities was kept confidential, and data will be anonymized.

**Ethical Approval**: The study protocol was reviewed and approved by the Institutional Review Board (IRB) of Faculty of Physical Education, Mansoura University.

**Pilot Study**

Pilot study carried on 30 women to test the objectivity and applicability of the research tools and the feasibility of the research process. Participants in the pilot study will be excluded from the research study. The pilot study revealed the feasibility, effectiveness, and appropriateness of the study instruments.
