# Supplementary material for: The effect of home-based yoga exercises on quality of life among postpartum women
Source: Reprod Health. 2026 Jun 13;23:114. doi: 10.1186/s12978-026-02357-2 (PMC13264819; doi:10.1186/s12978-026-02357-2)
Supplement: Supplementary file 2 — Supplementary Material 2. [file 12978_2026_2357_MOESM2_ESM.docx]

**Response to Reviewers**

**Manuscript Title:** *The Effect of Home-Based Yoga Exercises on Quality of Life among Postpartum Women*
**Manuscript ID:** [Add ID]

Dear Editor and Reviewers,

We would like to sincerely thank the reviewers for their valuable, insightful, and constructive comments. We have carefully revised the manuscript accordingly, and all changes have been highlighted in the revised version. Below is a detailed point-by-point response.

**Response to Reviewer 1**

**Comment 1: Writing style and language issues**

Numerous spelling, punctuation, and formatting errors affect readability.

**Response:**
Thank you for this important comment. The manuscript has been thoroughly revised for language accuracy, grammar, punctuation, and formatting. A professional academic editing process was conducted to ensure compliance with the journal’s guidelines and to enhance readability and clarity throughout the manuscript.

**Comment 2: Insufficient literature in introduction**

The introduction lacks depth and relies on limited references.

**Response:**
We appreciate this observation. The Introduction section has been substantially strengthened by incorporating recent and high-quality studies (2020–2025). We have avoided general statements and ensured that each claim is supported by multiple relevant references. Additionally, a more critical synthesis of the literature has been included to better justify the study rationale.

**Comment 3: Raw data tables unclear**

Tables are not well explained and difficult to interpret.

**Response:**
Thank you for highlighting this issue. All tables have been revised to improve clarity and presentation. Specifically:

- Clear titles and legends have been added.
- Abbreviations and symbols are now fully explained below each table.
- Raw data has been reorganized into summarized, reader-friendly formats.
- Explanatory text has been added in the Results section to guide interpretation.

**Comment 4: Weak discussion and comparison with literature**

Limited comparison with previous studies and lack of depth.

**Response:**
We have significantly enhanced the Discussion section by:

- Adding comparisons with multiple recent international studies.
- Clearly highlighting similarities and differences between findings.
- Providing possible explanations for the observed results.
- Expanding the discussion of the study’s contribution to postpartum care and maternal health.

**Comment 5: Missing strengths and limitations**

No clear discussion of study strengths and limitations.

**Response:**
Thank you for this valuable suggestion. A new subsection titled **“Strengths and Limitations”** has been added. This section discusses:

- Study strengths (e.g., home-based intervention, practical applicability).
- Limitations (e.g., sample size, self-reported data, short follow-up duration).
- Implications for generalizability.

**Comment 6: Lack of future recommendations**

No recommendations for future research.

**Response:**
We have revised the Conclusion section to include clear recommendations for:

- Future research directions.
- Integration of yoga-based interventions in postpartum care programs.
- Further investigation with larger and more diverse populations.

**Final Comment**

The study requires major revision.

**Response:**
We appreciate this overall assessment and have carefully addressed all concerns. We believe that the manuscript has been substantially improved in terms of scientific rigor, clarity, and presentation.

**Response to Reviewer 2**

**Comment 1: Manuscript needs paraphrasing**

**Response:**
Thank you for your comment. The manuscript has been extensively paraphrased to improve clarity, coherence, and academic tone while ensuring originality and avoiding redundancy.

**Comment 2: Role of nurses in the intervention is missing**

**Response:**
We appreciate this insightful suggestion. The Introduction and Discussion sections have been revised to clearly emphasize the role of nurses in:

- Educating postpartum women about home-based yoga exercises
- Supporting adherence and follow-up
- Promoting holistic postpartum care
- Integrating non-pharmacological interventions into nursing practice

This addition strengthens the relevance of the study within maternal and reproductive health nursing.

We sincerely thank the reviewers and the editor for their constructive feedback, which has significantly improved the quality of our manuscript. We hope that the revised version meets the journal’s expectations and is suitable for publication.

Sincerely,
[Doaa Mostafa Sheashaa]
On behalf of all authors
