# Supplementary material for: The effect of home-based yoga exercises on quality of life among postpartum women
Source: Reprod Health. 2026 Jun 13;23:114. doi: 10.1186/s12978-026-02357-2 (PMC13264819; doi:10.1186/s12978-026-02357-2)
Supplement: Supplementary file 3 — Supplementary Material 3. [file 12978_2026_2357_MOESM3_ESM.docx]

**Response to Reviewers**

Dear Editor,

We would like to express our sincere appreciation to you and the reviewers for the careful evaluation of our manuscript entitled:

**“The Effect of Home-Based Yoga Exercises on Quality of Life among Postpartum Women.”**

We are grateful for the constructive comments and valuable suggestions, which have helped us significantly improve the quality, clarity, and scientific rigor of our work. We have revised the manuscript accordingly and provide a detailed point-by-point response below.

**Reviewer Comments and Responses**

**Comment 1:**

*Within the first 6 months postpartum, what were the inclusion criteria for caesarean section?*

**Response:**

Thank you for this important comment. The inclusion criteria for women who had undergone cesarean section have now been clearly specified in the revised manuscript. Participants were included only if they:

- Had completed at least **6 weeks postpartum**
- Obtained **medical clearance from their healthcare provider**
- Had **no postoperative complications**, such as wound infection, severe pain, or delayed healing

This information has been added to the **Methods section**.

**Comment 2:**

*Is it appropriate for participants to practice yoga during the recovery period? Provide a clinical explanation.*

**Response:**

We appreciate this valuable suggestion. A clinical justification has now been incorporated into the manuscript. Postpartum yoga is generally considered safe when initiated after an appropriate recovery period (typically after 6 weeks postpartum for both vaginal and cesarean deliveries), and following medical approval.

Clinically, postpartum yoga has been shown to:

- Improve pelvic floor and abdominal muscle strength
- Reduce lower back pain and physical discomfort
- Enhance psychological well-being by reducing anxiety and depressive symptoms
- Promote circulation and overall physical recovery

Relevant and recent literature supporting these benefits has been added to the **Discussion section**.

**Comment 3:**

*The content of the Structured Interview Questionnaire must be specified.*

**Response:**

Thank you for this observation. The Structured Interview Questionnaire has now been described in detail in the **Methods section**. It includes:

- Socio-demographic data (age, education level, occupation, etc.)
- Obstetric and medical history (parity, mode of delivery, complications)
- Infant-related characteristics

This clarification improves transparency and reproducibility.

**Comment 4:**

*If it is a qualitative tool, explain the method of analysis.*

**Response:**

We would like to clarify that the Structured Interview Questionnaire is a **quantitative structured data collection tool**, not a qualitative instrument. Therefore, no qualitative analysis methods were used. This has now been explicitly stated in the revised manuscript.

**Comment 5:**

*Provide Cronbach’s alpha for the Quality of Life Questionnaire.*

**Response:**

Thank you for highlighting this important methodological aspect. The reliability of the Quality of Life Questionnaire has now been addressed as follows:

- The **Cronbach’s alpha reported in previous validation studies** has been cited
- The **Cronbach’s alpha calculated for the current study sample (α = XX)** has been added

This information is now included in the **Methods section under validity and reliability**.

**Comment 6:**

*The limitations of the study must be outlined.*

**Response:**

We appreciate this valuable suggestion. A dedicated **Limitations section** has now been added to the manuscript. The following limitations are acknowledged:

- Use of purposive sampling, which may limit generalizability
- Single-center study design
- Reliance on self-reported data, which may introduce reporting bias
- Short follow-up period, limiting assessment of long-term effects
- Lack of objective clinical outcome measures

This section has been incorporated into the **Discussion**.

**Conclusion**

We believe that the revisions made in response to the reviewers’ comments have substantially strengthened the manuscript. We sincerely appreciate the reviewers’ insightful feedback and hope that the revised version meets the standards for publication.

Thank you for your consideration.

Kind regards,
**Dr. Doaa Mostafa Sheashaa**
On behalf of all authors
